# Towards a risk assessment framework for micro- and nanoplastic particles for human health

**DOI:** 10.1186/s12989-024-00602-9

**Published:** 2024-11-29

**Authors:** Amelie Vogel, Jutta Tentschert, Raymond Pieters, Francesca Bennet, Hubert Dirven, Annemijne van den Berg, Esther Lenssen, Maartje Rietdijk, Dirk Broßell, Andrea Haase

**Affiliations:** 1 Department of Chemical and Product Safety, German Federal Institute for Risk Assessment (BfR), Berlin, Germany; 2 Institute for Risk Assessment Sciences, Utrecht University, Utrecht, The Netherlands; 3 Federal Institute for Materials Research and Testing (BAM), Berlin, Germany; 4 Department of Environmental Health, Norwegian Institute of Public Health (NPIH), Oslo, Norway; 5 Amsterdam UMC, Amsterdam, The Netherlands; 6 Federal Institute for Occupational Safety and Health (BAuA), Berlin, Germany; 7 Institute of Pharmacy, Freie Universität Berlin, Berlin, Germany

**Keywords:** Microplastics, Nanoplastics, Human health, Risk assessment, Integrated approaches to testing and assessment (IATAs), Polymers of low concern (PLC), Poorly soluble low toxicity particles (PSLT)

## Abstract

**Background:**

Human exposure to micro- and nanoplastic particles (MNPs) is inevitable but human health risk assessment remains challenging for several reasons. MNPs are complex mixtures of particles derived from different polymer types, which may contain plenty of additives and/or contaminants. MNPs cover broad size distributions and often have irregular shapes and morphologies. Moreover, several of their properties change over time due to aging/ weathering. Case-by-case assessment of each MNP type does not seem feasible, more straightforward methodologies are needed. However, conceptual approaches for human health risk assessment are rare, reliable methods for exposure and hazard assessment are largely missing, and meaningful data is scarce.

**Methods:**

Here we reviewed the state-of-the-art concerning risk assessment of chemicals with a specific focus on polymers as well as on (nano-)particles and fibres. For this purpose, we broadly screened relevant knowledge including guidance documents, standards, scientific publications, publicly available reports. We identified several suitable concepts such as: (i) polymers of low concern (PLC), (ii) poorly soluble low toxicity particles (PSLT) and (iii) fibre pathogenicity paradigm (FPP). We also aimed to identify promising methods, which may serve as a reasonable starting point for a test strategy.

**Results and conclusion:**

Here, we propose a state-of-the-art modular risk assessment framework for MNPs, focusing primarily on inhalation as a key exposure route for humans that combines several integrated approaches to testing and assessment (IATAs). The framework starts with basic physicochemical characterisation (step 1), followed by assessing the potential for inhalative exposure (step 2) and includes several modules for toxicological assessment (step 3). We provide guidance on how to apply the framework and suggest suitable methods for characterization of physicochemical properties, exposure and hazard assessment. We put special emphasis on new approach methodologies (NAMs) and included grouping, where adequate. The framework has been improved in several iterative cycles by taking into account expert feedback and is currently being tested in several case studies. Overall, it can be regarded as an important step forward to tackle human health risk assessment.

**Supplementary Information:**

The online version contains supplementary material available at 10.1186/s12989-024-00602-9.

## Background

The interest in micro- and nanoplastic particles (MNPs) has risen considerably in the last years due to increasing public awareness of plastic contamination across the globe and insufficient knowledge about potential human health hazards [1, 2]. Due to many advantageous properties, plastic production has increased from 5 million metric tons globally in the 1950s to 400 million tons in 2022 [3]. Mismanaged plastic waste contributes significantly to the overall plastic pollution, which has been predicted to triple towards 2060 if no urgent measures are taken [4]. Plastic pollution was identified as one of the most serious environmental challenges of our century [5].

The terms microplastics (MPs) and nanoplastics (NPs) are generally not well defined and different terminologies have been suggested [6]. MPs are typically understood as solid plastic particles being smaller than 5 mm [7]. NPs are often understood in analogy to nanomaterials as solid plastic particles with one or more external dimensions in the size range of 1–100 nm [8]. However, also an upper size limit of 1000 nm has been suggested [9]. Hartmann et al. [6] proposed a comprehensive framework for defining and categorizing plastic debris taking into account four classifiers, namely origin, size, shape and color. In terms of their origin, plastic particles can be categorized as primary or secondary. Primary MPs are intentionally manufactured for a specific purpose such as pellets for plastic productions or abrasive beads, which can be released into the environment due to unintentional spills during production, transport or disposal. Secondary MPs result from the degradation of larger plastic items such as plastic bags or bottles. The degradation process is facilitated by various environmental factors, including UV light, heat or mechanical processes including wave abrasion. It is estimated that the majority of MPs in the environment is secondary. Tire wear particles were identified as a major source [10] with estimated releases reaching 1,327,000 tonnes each year, in the EU alone [11]. They are mostly derived from traffic-related abrasion but may also occur from crumb granulates of recycled tires, which is used, for instance, as infill materials for sports fields [12].

Due to their high biopersistence, MPs are widespread in the environment. Hence, they have been detected in various types, sizes and shapes/ morphologies in virtually every environmental compartment, including indoor and outdoor air [13–15], the marine environment [16] and sediments [17]. The most frequent shapes include fibres, fragments, and films. MPs have also been detected in various food items, including sea food, table salt or honey, as well as in drinking water and other beverages, as summarized by Touissant et al. [18]. However, methods for reliable detection and in particular quantification, especially in complex matrices, are still under development. Due to their widespread occurrence, humans can be exposed to MPs via different exposure routes with ingestion and inhalation generally regarded as the most prominent [19]. Dermal exposure has also been discussed but seems not as relevant as MPs cannot penetrate the intact human skin [20]. Nor and co-workers established a toolkit for estimating the lifetime accumulation of MNPs through ingestion and inhalation, and suggested a daily intake of 553 particles per day for children and 883 particles per day for adults [21]. Indeed, MPs were not only detected in human stool but also in human tissues such as lung, placenta and, most recently, in carotic plaques [22–27]. Evidence for systemic bioavailability is increasing, and hence research has broadened to address potential adverse effects on humans [28, 29]. The World Health Organization (WHO) published a very comprehensive report on possible human health implications and evaluated many in vivo and in vitro studies [30]. Overall, the described effects for MNPs were similar to those known from other solid, insoluble particles. However, WHO also emphasized many limitations which are discussed in the next chapter. Overall, human health risk assessment of MNPs is still in its very infancy. However, the evidence for environmental risks was considered substantial enough for a first regulatory measure in the EU in 2023 with the amendment of EU REACH to restrict specific types of synthetic polymer microparticles [31].

It should be noted, that most research so far focused on MPs only. NPs only recently gained more attention. Research on NPs is much more challenging as analytical and sampling methods are largely lacking. For instance, particle extraction from environmental matrixes is limited by the mesh sizes of nets or sieves. However, as the majority of plastics debris in the environment are secondary in origin, it might be reasonable to assume that the degradation process would also give rise to smaller (i.e. nanoscaled) particles. It also should be kept in mind that the smaller particles may outreach the larger ones significantly by number even when their overall mass might be very low. For MPs it was already shown that they substantially outnumber larger plastic items in marine systems but account only for a small proportion of the total plastic mass in the ocean [32, 33]. This might be equally true for NPs when compared to MPs.

In the following we will consistently use the term MNPs to collectively refer to micro- and nanoscaled plastic particles but it should be kept in mind that existing knowledge mainly covers MPs. Our aim was to propose a human health risk assessment framework for MNPs. This work has been conducted within the EU funded project POLYRISK (ID: 964766, webpage: www.polyrisk.science), where the primary focus was on inhalative exposure. To develop the framework state-of-the-art knowledge was reviewed, considering different legal frameworks for chemical safety, relevant guidance documents for risk assessment, standards, scientific publications as well as project reports. Furthermore, we also comprehensively reviewed existing test guidelines, methods and tools to suggest promising ones as reasonable starting points. In the following we firstly summarize the existing knowledge and next, use suitable elements to construct the modular POLYRISK risk assessment framework for MNPs.

## Towards a human health risk assessment framework for MNPs

### The general risk assessment paradigm

Chemical risk assessment always starts with identification and proper description of what is going to be assessed, e.g., a specific chemical, a group of chemicals or a mixture. In some cases, the unique identification is possible based on knowledge of the chemical composition alone, also taking into account impurities. In other cases, however, in addition specific physicochemical descriptors are required, as exemplified for nanoforms of substances under EU REACH. Chemical risk assessment is founded on two pillars, hazard and exposure assessment. The exposure to a chemical substance can be direct or indirect (if the chemical/ particle is contained in a product and firstly needs to be released). Hazard assessment includes hazard identification (e.g., which adverse effects are caused) and hazard characterization (e.g., the dose–response characterization).

### Risk assessment of MNPs

#### Challenges

Of course, the general risk assessment paradigm is equally applicable for MNPs. The first challenge is to properly describe the “item” which shall be assessed. MNPs represent a very heterogenous group of particles with a broad spectrum of physicochemical properties (e.g. sizes, shapes/ morphologies), which can be based on different polymer types, such as polyethylene (PE), polypropylene (PP), polyamide (PA), polyurethane (PU) or polystyrene (PS), but also may contain a variety of other chemicals, including plenty of additives. In 2016, ECHA has listed more than 400 additives that are used in plastic manufacturing [34]. It is estimated, that additives account on average for up to 4% (w/w) of the plastic weight, but this value is dependent on the polymer type and its use and is therefore highly variable [35]. Examples include different antioxidants, plasticizers or pigments. Some additives may have adverse health effects, for example, certain phthalates are endocrine disruptors [36]. In addition, there are numerous vulcanizing agents, vulcanization accelerators or activators and other additives that are used specifically in the production of tire rubber [37, 38]. Some of them have potential toxic properties, including benzothiazole which is a respiratory irritant and dermal sensitizer [39] or *N*-(1,3-Dimethylbutyl)-*N*′-phenyl-p-phenylendiamine (6PPD) which is toxic to reproduction [40]. Moreover, MNPs can contain residual monomers or oligomers from incomplete synthesis, some of which may also be toxic for humans. One frequently cited example is bisphenol A, a known endocrine disruptor [41]. However, bisphenol A represents only one example, other bisphenols may have similar toxic effects. Overall, the United Nations Environmental Programme has identified 10 groups of chemicals of major concern. Other examples for groups of major concern are per- and polyfluoroalkyl substances, phthalates, and metals [35]. In addition, plastic materials contain a high number of non-intentionally added substances (NIAS), which are substances that are not added for a technical reason. NIAS can have various sources, broadly categorized as side products, breakdown products, and contaminants. Side products may be formed during the production such as polymerization side products. In addition, several constituents (e.g., polymers) as well as additives can be degraded, during manufacturing and use, leading to a variety of possible breakdown products.

MNPs may also act as a carrier for various environmental pollutants, including polycyclic aromatic hydrocarbons (PAHs), polychlorinated biphenyls (PCBs) or heavy metals, also with well-known toxic properties. Studies on the so called “carrier effect” have mainly been conducted in an ecotoxicological context [42–44]. The overall conclusion is that MNPs can act as transport vehicles but the “carrier effect” is rather negligible, at least when compared to the overall exposure to these environmental contaminants [45]. Research on the “carrier effect” for human health is still scarce. Finally, the formation of a microbiological biofilm on the surface of MNPs may also lead to adverse effects. It has been hypothesized that this biofilm could trigger some immune responses or induce changes in the gut microbiota [46]. However, research into biofilms is still in its very infancy.

Finally, due to aging and weathering, several physicochemical properties will change over the life cycle, which may affect risk assessment and hence should be considered.

Other challenges arise from available data and the question whether this data is appropriate. For instance, there is a discrepancy between the MNPs size range that can be detected in the environment and the size range being investigated in research [30]. Most studies investigate particles smaller than 1 µm but due to analytical challenges so far mostly larger particles have been detected and quantified in the environment. Moreover, most toxicity studies investigate monodisperse spherical PS beads (67% of the evaluated studies in the WHO report) as these particles are readily commercially available in different sizes and with surface treatments including fluorescent labels. Most of toxicity studies focused on oral exposure while studies concerning the inhalation route are only recently emerging. Health Canada concluded: “The current literature on the human health effects of microplastics is limited, although a concern for human health has not been identified at this time” and “while some occupational epidemiology and experimental animal studies show the potential for effects at high exposure concentrations, they are of questionable reliability and relevance, and further research on the potential for microplastics to impact human health is required” [47].

The overarching challenges for MNPs risk assessment are lack of reliable data and reliable methods. Overall, data is very scarce and its relevance and reliability is often questionable. For instance, most toxicological studies on inhalation that were evaluated in the WHO report were found to be inadequate in terms of the quality assurance and quality control (QA/QC) criteria proposed by Gouin et al. [48]. These QA/QC criteria include important aspects of particle characterization, the experimental study design and the applicability for risk assessment. In addition, methods that are specifically developed or adapted for MNPs are largely lacking. In particular, analytical methods for detecting NPs remain challenging. Generally speaking, methods for reliable quantification in complex matrices are still in development. For hazard characterization, methods being developed for nanomaterials are often applied but uncertainties remain as several adaptations might be needed. MNPs often show a much broader size distribution. Smaller particles are of higher concern due to a higher likelihood of uptake and transport across barriers but their effects might not be detected when they represent only a tiny mass fraction, especially during the typical short observation times in vitro. Another critical aspect could be that MNPs, depending on their density, might not sediment and reaches cells cultivated in vitro at the bottom of plastic dishes.

#### State-of-the-art and existing frameworks

A few comprehensive reports, released by the European Food Safety Authority (EFSA), Health Canada and the World Health Organization (WHO) [30, 47, 49], summarize the state-of-the-art of MNP risk assessment and describe key challenges, which were summarized above. To date, there are only a few published frameworks for risk assessment and/or risk management of MNPs. Most are designed for environmental risk assessment [50–52]. A few also provide tools that can be useful for human health risk assessment [53–57]. To the best of our knowledge, there are only two comprehensive approaches for risk assessment that specifically address human health risks of MNPs [58, 59]. The framework proposed by Nor and co-authors was designed for regulatory use and in principle follows the classic risk assessment paradigm described above [58, 60]. Due to the complexity of the risk assessment of MNPs it describes four pillars that address different needs: (I) analytical techniques, (II) empirical data, (III) theoretical and modeling approaches and (IV) stakeholder engagement. One important hallmark of this framework is the implementation of probability density functions (PDFs). PDFs are mathematical functions that can be applied to define the probability of a variable (for example a certain physicochemical property of MNPs). In the absence of precise data, PDFs for MNPs characteristics can be a valuable tool that also properly captures the complexity and heterogenicity of MNPs. So far, PDFs have been established for size, shape and density of MNPs [54, 56]. For example, Kooi and Koelmans investigated nineteen particle size distributions from 11 studies and found two different patterns [54]. Either the studies showed a decrease in particle concentration with an increase in size, or they found an initial increase in concentration with particle size, followed by a decrease similar to the first pattern. The authors also provide some reasoning on these patterns. However, their analysis allowed them to establish a generic continuous particle size distribution with lower and upper boundaries of 20 μm and 5 mm, respectively. Similarly, they have done an analysis of shape and density distributions. For shape, the most abundant categories in water and sediment were fibers (48.5%), followed by fragments (31%), beads (6.5%), films (5.5%), and foam (3.5%), which allowed them to derive a continuous bimodal microplastic shape distribution. Another approach for the development of a human health risk assessment framework has been proposed by Christopher et al. which specifically addresses developmental toxicity and impacts of MNPs on early-life health. For this purpose, the authors take into account classical risk assessment approaches and systematically elucidate different stages of classical risk assessment in the context of MNPs [59]. In addition, the authors identified current knowledge and research gaps for MNPs. These include, among others, standardized reporting, reference materials and the application of already established paradigms or concepts (for instance, those that are used for nanomaterials).

Another risk assessment framework for MNPs has been proposed by Bucci and Rochman, which is a more general framework, not explicitly established for human health [53]. Here, the key element is a scoring system that is used to calculate and predict the hazard of MNPs in the environment. In the proposed framework, different scores are given within four different categories, namely size, shape, polymer type and environmental chemistry, which relates to the level of contamination. The scores range from 0.1 to 0.9, with 0.9 being the highest hazard. The different categories are then combined. The suggested ranking is mainly based on the work of Lithner et al. [61]. Within this scoring system, “particle-specific hazard values” are determined for the different types of particles. Once these particle-specific hazard values are determined, the authors suggest to multiply the values by the number of the corresponding particle types in the environmental sample. However, an exposure assessment is generally not considered within this framework, which is certainly a critical point. Furthermore, the real relevance or meanings of such calculated scores remains highly questionable (Table 1).

**Table 1:** Scoring system to predict and calculate the hazard of MNPs in the environment proposed by Bucci and Rochman [53], table with minor adaptations

Ranking	Size	Shape	Polymer type	Environmental chemistry
0.1	> 1 mm	Sphere	PP, polyvinyl acetate, cellulose	Pristine or relatively clean water body
0.2				
0.3	0.1–0.9 mm (100–999 µm)		PS, LDPE, HDPE, polyethylene terephthalate	
0.4	0.01–0.09 mm (10–99 µm)			
0.5			PA, expanded PS	Moderately polluted water body
0.6		Fragment		
0.7	0.001–0.009 mm (1–9 µm)		Polycarbonate, polymethylmethacrylate	
0.8				
0.9		Fibre	PVC, polyurethane, rubber	Highly polluted water body (e.g. wastewater effluent, highly populated, industrial, agricultural areas)

#### General recommendations and future needs

To enable a thorough risk assessment of MNPs, experts have proposed several recommendations to address current challenges [47, 50, 58, 62, 63]. These recommendations cover different aspects of exposure and hazard assessment. In addition, the consideration of legal frameworks for chemical safety and existing concepts for particle and fibre toxicology might be helpful for the risk assessment of MNPs. It is also crucial to implement methodologies that are appropriate, particularly in regard to risk assessment.

##### Sampling, extraction and detection

Recommendations for the sampling and the analysis of MNPs have been summarized in a previous WHO report [64]. Although this report addresses MPs in drinking water, the recommendations are equally valid for other matrices. For instance, methods (e.g., for sampling, extraction and detection) should always be described in detail and their reproducibility should be demonstrated. It is considered very important to standardise the methods before they are applied for risk assessment. For example, data obtained for specific physicochemical properties are very much dependent on the applied method, emphasizing the need for standardized procedures. In addition, reference materials are important, for instance, for the validation of analytical methods.

To prevent MP contamination, it is important to clean and rinse laboratory surfaces with filtered water. It is also necessary to implement positive controls to determine recovery after digestion, density separation, and filtration steps. Blank samples or filters should also be included to get information on the background of particles.

##### Physicochemical properties and dosimetry

Furthermore, as also known from nanomaterials, it is important to characterise the physicochemical properties also in the context of in vitro and in vivo studies in the appropriate biological fluids as several properties of the particles can change over time in different matrices. This includes properties such as size and size distribution, but should also include surface chemistry and surface reactivity. The determination of the effective particle density could be relevant for dosimetry models. The in vitro dosimetry models are explained later. One relevant in vivo dosimetry model is the multi-path particle dosimetry (MPPD) model from the US EPA, which calculates the deposition of particles from aerosols in the different regions of the respiratory tract [65]. Such models can also be very useful for MNPs exposure assessment. In general, the use of in silico models can be helpful. Unfortunately, so far only very few exist for MNPs. One notable example are the aforementioned PDFs [54, 57].

##### Toxicological assessment

For in vitro and in vivo investigations current limitations are the lack of variety of relevant test materials as mostly PS has been tested in the available studies. For a comprehensive hazard assessment, a broader selection of MNPs, including different sizes (also polydisperse materials), shapes or polymer types, are recommended, and benchmark materials should be established. In addition, reliable methods that are specifically applicable for (polydisperse) MNPs are urgently needed. As previously mentioned, guidance on QA/QC criteria has already been provided for MNPs [48]. In addition, there are well-founded general recommendations on how to establish scientific credibility in new approach methodologies (NAMs) [66].

##### Risk assessment

The existing knowledge on particle toxicology in general, and inhalation toxicology in particular, should be implemented for the risk assessment of MNPs [67]. Humans can be exposed to a broad variety of natural and synthetic particles, which may well share common modes of actions. In this regard, it has been stated that none of the individual properties of MNPs are unique per se. It rather is the combination of certain characteristics that make MNPs unique and/or potentially hazardous. A specific aspect for the risk assessment of MNPs is to consider the leaching of additives, contaminants and residual monomers/oligomers.

It is evident that risk assessment of MNPs cannot be achieved on a case-by-case basis, considering all possible particle types/ variants in combination with all the possible additives and environmental contaminants and moreover taking into account different stages of aging/weathering. Other, more straightforward approaches are required. Several existing concepts and approaches may be applied and/or adapted for this purpose. For instance, within EU REACH there is a general guidance for substances of Unknown or Variable composition, Complex reaction products or Biological materials (UVCB), which may serve as a good starting point for the classification/identification of complex MNPs mixtures [68]. According to this guidance document, UVCBs are characterized to contain a relatively large number of constituents, their composition might be (to a significant part) unknown and/or the composition could be variable or poorly predictable. This guidance recommends to describe the chemical composition and the identity of the constituents as far as known, which could also be done in a generic manner. Furthermore, it is recommended to describe the source and the specifications of the process, which partially can be applied as well for MNPs.

Another example is the risk assessment of non-intentionally added substances (NIAS) in food contact materials, which is equally challenging. Food contact materials may contain plenty of intentionally added chemicals, some of which are already quite complex such as printing inks being chemical mixtures of pigments, solvents, monomers, photoinitiators and other compounds. Contaminants may originate from different sources. Often these are environmental contaminants, which may remain in the final product in traces. Additionally, contaminants can be process-related, e.g. being introduced via recycling. Overall, the variety of NIAS is enormous huge, some of which are known but many are unknown. Their amounts can also vary significantly. Furthermore, their profiles will change over time, e.g., due to oxidation or other forms of aging. Tiered approaches have been proposed to tackle NIAS risk assessment. They generally start with an analytical screening of leachates. Similar approaches could be adopted and integrated into the risk assessment of MNPs, specifically to consider additives and/or contaminants.

In summary, a human health risk assessment of MNPs is challenging and complex. Reliable concepts are urgently needed, taking into account the ever-increasing plastic pollution, the biopersistence of MNPs and potential adverse effects which might be derived from similarities to other solid, persistent particles. There is plenty of existing knowledge, which can serve as a good starting point to establish a human health risk assessment framework for MNPs.

### The POLYRISK risk assessment framework for MNPs

The POLYRISK risk assessment framework aims to support human health risk assessment of MNPs with a primary focus on inhalation as one of the most relevant exposure routes for humans. To develop the framework, state-of-the-art knowledge was reviewed, considering different legal frameworks, relevant guidance documents, standards, scientific publications as well as project reports. We identified suitable elements and building blocks of the existing approaches and assemble them into a modular risk assessment framework using the concept of integrated approaches to testing and assessment (IATAs). The POLYRISK risk assessment framework is modular and consists of several consecutive steps, starting with basic physicochemical characterization of the material (step 1) in order to investigate whether the sample meets the criteria for MNPs. This is followed by step 2, the consideration of several parameters that are relevant to assess whether the particle can be inhaled. Finally, several in vitro testing strategies that are applicable to different morphologies of MNPs are proposed in step 3. They include an IATA to test the fibre grouping hypothesis (step 3.1) and an IATA to test for poorly soluble low toxicity (PSLT) particles (step 3.2). For each step we suggest test methods, which were carefully selected. Importantly, the framework is applicable for both primary MNPs, which are specifically produced as well as for secondary MNPs, as sampled from the environment. As the framework is modular, it can be easily amended (as needed) and we already suggest several options to extend the framework. This applies, for example, to other exposure routes or the investigation of the leaching of additives/contaminants.

Our framework represents a simplified but practical approach which mainly aims for classification and prioritization. Therefore, we also implemented several grouping approaches, where applicable. To establish the framework, we considered the state-of-the art knowledge on adverse outcome pathways (AOPs).

### Integrated approaches to testing and assessment (IATA)

An IATA is a flexible approach for chemical safety assessment that “integrates and weights all relevant existing evidence and guides the targeted generation of new data, where required, to inform regulatory decision-making regarding potential hazard and/or risk. Within an IATA, data from various information sources (i.e. physicochemical properties, in silico models, grouping and read-across approaches, in vitro methods, in vivo tests and human data) are evaluated and integrated to draw conclusions on the hazard and/or risk of chemicals” [69].

In order to establish an IATA, knowledge on specific adverse outcome pathways (AOPs) can be very helpful, as explained below. Recently several IATAs have been proposed by the EU GRACIOUS project (ID: 760840) to substantiate grouping and read-across of nanomaterials (including nanofibers). In addition, the US EPA proposed an IATA to group poorly soluble polymer particles [70]. They will be discussed in the following sections in more details as they have been implemented (as far as possible) into the POLYRISK risk assessment framework.

### Grouping and read-across

Grouping and read-across are the most commonly applied alternative approaches to animal testing in regulatory risk assessment. Generally speaking, more than one chemical is considered simultaneously thereby reducing the time and the resources required for testing [71, 72]. Chemicals can be categorized in a group, if their “physicochemical, toxicological and ecotoxicological properties are likely to be similar or follow a regular pattern as a result of structural similarity” [73]. Within an established group read-across can be applied to predict an endpoint for one chemical (target) by using existing data of other chemical(s) (source material(s)). Grouping and read-across approaches are useful to fill in data gaps without the need for new animal tests. For regulatory acceptance of grouping it is mandatory to provide a justification, e.g. a scientifically sound hypothesis that links specific physicochemical properties with specific hazards of a chemical substance [74].

Several grouping approaches for nanomaterials have been already established, with the most comprehensive and recent one released by the EU project GRACIOUS [75].

### Adverse outcome pathways (AOP)

The AOP concept was originally described by Ankley and co-workers as conceptual constructs [76]. AOPs integrate known information from various sources into a sequential chain of causally linked key events (KEs) occurring on different biological levels (i.e., cellular, tissue, organ level) [76], starting with a molecular initiating event (MIE) leading to the final adverse outcome (AO) [77]. AOPs can support risk assessment by organizing the existing knowledge. IATAs are often built on existing AOPs with various tests that each address different KEs [78]. A comprehensive catalogue of the currently existing AOPs is available on the AOP-Wiki website https://aopwiki.org (version 2.7., March 2024).

The AOP concept has been further evolved in the context of the OECD. Within the OECD Working Party of Manufactured Nanomaterials a project on ‘Advancing Adverse Outcome Pathway Development for Nanomaterial Risk Assessment and Categorization’ was conducted with the aim to identify KEs from the existing nanotoxicology literature [79]. As a result, several AOPs with a particular focus on lung toxicity, were endorsed to be also relevant for NMs, namely AOP 173, AOP 237, AOP 302, and AOP 303 [80]. For instance, AOP 303 deals with frustrated phagocytosis leading to lung cancer. The most frequently reported KEs for nanomaterials are “cytotoxicity”, “reactive oxygen species (ROS) generation”, “oxidative stress” and “persistent inflammation”. Even though AOPs as such are compound agnostic, the MIE may differ for nanomaterials. Several initiating events were suggested to be relevant for nanomaterials: (i) interaction of particles/fibres with cell membranes/biomolecules, (ii) reactive oxygen species (ROS) formation/generation, (iii) lysosomal injury/damage/disruption, (iv) DNA damage/methylation, and (v) inflammation induction [81].

**Fig. 1:**
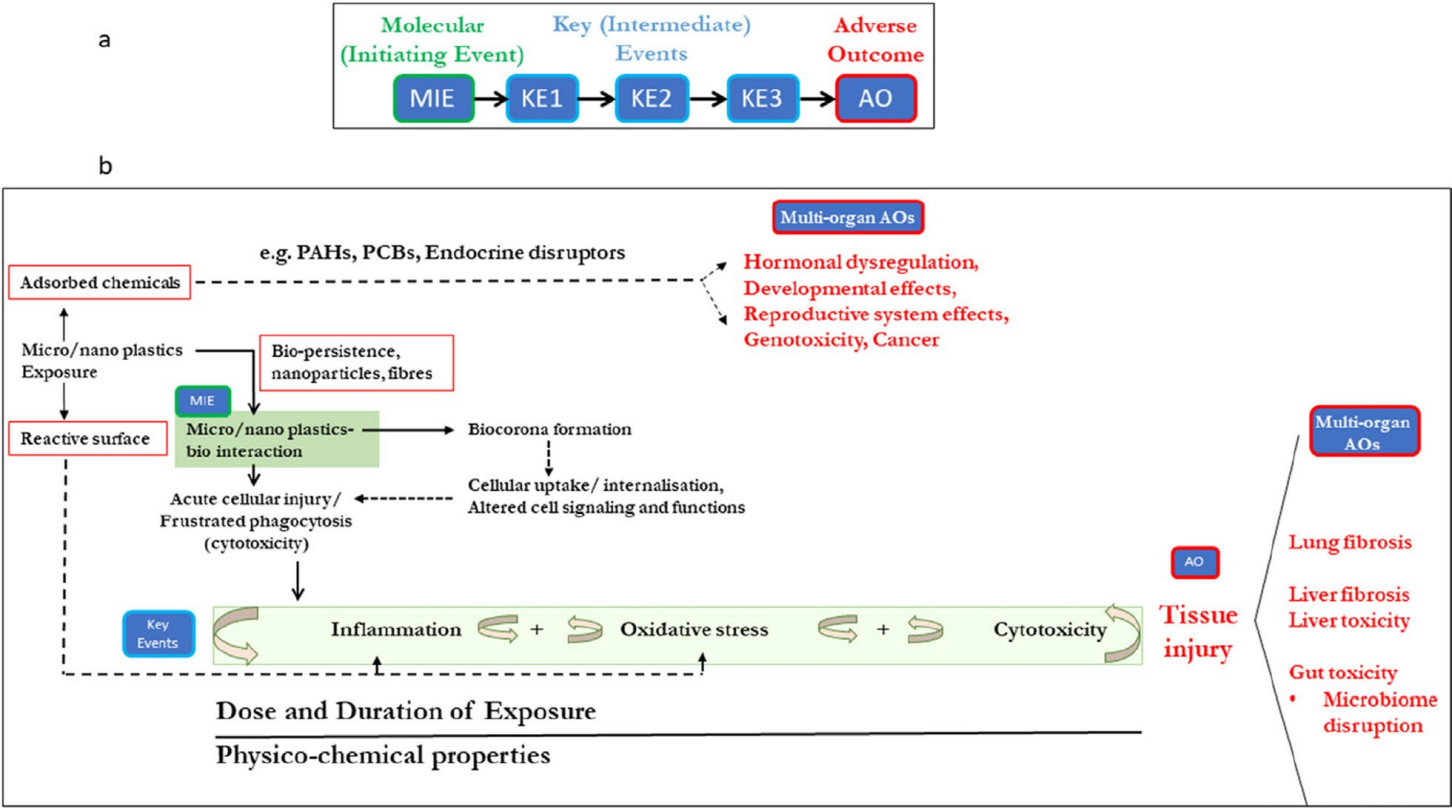
Mini-AOP for microplastics proposed by Halappanavar and Mallach [62]

In addition, a few researchers have started to identify relevant AOPs for MNPs. Some also considered selected additives which may leach from the particles. ROS generation has been suggested as an initiating event for MNPs [82]. Common KEs for MNPs were “inflammation”, “oxidative stress” and “cytotoxicity” [82–84]. Wright and Borm stated that MIEs for different particle species may differ [85]. Halappanavar and Mallach have proposed a “Mini-AOP” specifically for MPs which is shown in Fig. 1 [62]. The proposed MIE is the interaction between the particle and the cell membrane, as described in AOP 173 for lung fibrosis after particle inhalation. The KEs are inflammation, oxidative stress, and cytotoxicity, which are in good agreement with the aforementioned findings. It should be noted, that “inflammation” is a complex biological process that may include different KEs such as the release of pro-inflammatory mediators (e.g. cytokines) or the recruitment of specific cell types (e.g., leukocytes) to the site of inflammation. Also, inflammation and oxidative stress are often linked [86]. Moreover, inflammation is also important for tissue homeostasis, which is not reflected by this Mini-AOP [87].

Additionally, a second AOP specifically for MNP exposure via oral ingestion has been proposed by Jones et al. [88]. Their findings on KEs after MNP exposure are largely consistent with the “Mini-AOP” described above. In addition, to the KEs “inflammation” and “oxidative stress” the authors suggest “effects on lipid metabolism” and “amino acid and energy metabolism” to be relevant. The authors emphasise that there is currently no known adverse effect of MNPs.

Furthermore, relevant toxicity mechanisms of 50 selected additives have been investigated using the database ToxCast™ to systemically evaluate the toxicity of the different additives [84]. The authors suggest, that inflammation, effects on lipid metabolism, neurotoxicity and KEs that could lead to cancer may be the most prominent KEs or assay endpoint identifier linked to chemical additives of MNPs.

In summary, several MIEs and KEs have been identified and proposed to be relevant for MNPs. Importantly, an AO is still lacking. We considered the knowledge on the selected AOPs for the establishment of the POLYRISK risk assessment framework and incorporated the proposed KEs.

In addition, we also identified the following relevant concepts that shall be briefly summarized below.

### Polymers of low concern (PLC) concept

MNPs are composed of numerous types of polymers, with PE, PP (both polyolefins) and polyvinyl chloride (PVC) being the most abundant. Hence, we also considered approaches for risk assessment of polymers. A pragmatic approach is based on the polymers of low concern (PLC) concept. The PLC concept has been applied by the United States Environmental Protection Agency (US EPA) and the Australian Industrial Chemicals Introduction Scheme (AICIS) since the mid 1990s, as well as by the Canadian Environmental Protection Act (CEPA) since 2005 [89]. In the EU, specific criteria have been introduced in 2020 for the identification of polymers requiring registration (PRR) under EU REACH. These criteria are based on a similar concept as the PLC criteria [90]. Overall, the PLC concept identifies criteria which allow the categorization of polymers as either PLC or non-PLC. However, the criteria can differ in detail between agencies and countries.

Common criteria for considering a polymer as a PLC were summarized by an OECD expert group [91]. Firstly, the number-average molecular weight (M_n_) of a polymer being ≥ 1000 Da. This is the most commonly used criterion for identifying a PLC. This is based on the assumption that the smaller the molecule is, the easier it crosses a cellular membrane. Secondly, the amount of low molecular weight oligomeric species contained in a polymer should not exceed a certain level. In general, it is proposed that this should not be more than 5% of < 1000 Da oligomeric species and more than 2% of < 500 Da oligomers. Thirdly, a further significant criterion is the absence or presence of reactive functional groups (RFGs). RFGs contain cationic species that are known to cause aquatic environmental toxicity, and which may therefore contribute to the toxicity of these polymers. However, the available data on RFG was insufficient to determine the level of concern for any RFGs in the PLC criteria. Other criteria which can be used for the classification of a PLC are, for example, solubility in water and other solvents (polymers with a water solubility < 10 mg/L showed generally low health concern), the polymer type (chemical class) or the residual monomer content.

In 2009, an OECD working group published an evaluation of 205 different polymers taking into account the PLC criteria. According to this working group, PLCs are polymers which have “insignificant environmental health and human health impacts” and “therefore, these polymers should have reduced regulatory requirements” [91]. Different polymer types, molecular weight, contents of low weight oligomeric species and functional groups were considered in this study. Polymer types were categorized into 12 different classes: polyesters, polyolefins, polyacrylates, polyethers, polyurethanes, polyamides, polyimides, polysaccharides, polyvinyl, siloxanes and silicones, epoxy resins and “others”. Polymers with unique or uncertain characteristics were placed into the “others” category. Overall, 139 polymers were classified as PLCs  in this report according to the US EPA criteria. 87.8% of those PLCs showed low health or ecotoxicological concern according to available (eco)toxicological data that has been provided by the USA, Canada, Australia Japan and Korea. This indicates that the PLC criteria are applicable for the vast majority of polymers. Interestingly, the OECD working group found a clear trend between the polymer type and low or potential health concerns. In addition, it was noted that it remained unclear whether the toxicity of the PLCs which showed some potential for health concern was an artefact, or whether the toxicity of those was due to a mechanism, which is currently not covered by the PLC criteria. The most commonly identified reactive functional groups (RFG) for polymers were amino, epoxide, isocyanate and anhydride groups.

In general, the polymer types, which are of major relevance in the context of MNPs are all considered as PLCs [92]. When assessing MNPs in vitro, we recommend to characterize the particles for their *M*_*n*_ and their surface reactivity. It could be possible that not all the PLC criteria are met. Firstly, some nanosized polymer powders may have a *M*_*n*_ < 1000 Da. Secondly, weathering of MNPs might decrease the *M*_*n*_ [93]. In addition, weathering could alter the oxidation degree of the particle surface, as shown by several studies [94, 95]. Several functional groups, including ketone, aldehyde, acid halides, carboxylic acid groups were identified by Raman spectroscopy after weathering of PP and PE particles [96]. Aldehydes and acid halides are classified as moderate-concern functional groups according to USA EPA while all other identified groups in this study are generally classified as low-concern functional groups [97].

### Poorly soluble low toxicity particles (PSLT) concept

MNPs have been detected in both indoor and outdoor air samples, indicating their contribution to the overall particle pollution in the ambient air [13, 98–100]. The primary sources of atmospheric MNPs include tire abrasion, (the production of) textiles, and waste incineration [47, 101]. A study focusing on indoor environments, found that MPs made up 4% of the particles identified in three separate apartments. The other particles were identified as being “non-synthetic”. Overall, the median diameter (D_50_) of the MPs was calculated to be 36 µm. However, due to limitations in analytical techniques only particles larger than 11 µm were detected [98]. In a more recent study MPs < 5 µm were detected in a concentration between 58 and 684 particles per m^3^ depending on the location (one meeting room, one workshop and two apartments) using micro-Raman spectroscopy [102]. However, these concentrations seem to be very low, compared to the overall concentration of particulate matter (PM) in ambient air [103, 104]. Particulate matter in the air is classified according to the particle sizes. The PM_10_ fraction covers particulate matter in the air with a maximum diameter of 10 µm, the PM_2.5_ fraction has a maximum diameter of 2.5 µm while ultrafine particles have a maximum diameter of 100 nm. It should be noted, that reliable analytical methods for the detection of smaller polymer particles are currently still missing, and hence at the present time it is very difficult to estimate their contribution to PM. However, particularly for tire wear particles, a few attempts have been made to assess this contribution. For example, it has been estimated that tire wear particles contribute to approximately up to 8% of the PM_10_ fraction and 10% of the PM_2.5_ fraction [105]. In addition, an understanding of the contribution of MNPs to ambient PM might be important for the identification of the human health hazard potential. The WHO report from 2022 concludes that current data and evidence indicate that MNPs may have adverse effects similar to those of other solid particles, and furthermore, share similar modes of action [30]. A read-across approach could be therefore useful.

Whether or not a particle can be inhaled and how deep it can enter into the lung is not only defined by its diameter but by its aerodynamic diameter, which also takes into account the particle density, the flow velocity and the air viscosity. Hence, in particle inhalation toxicology the most important parameter is the mass median aerodynamic diameter (MMAD), which specifies the aerodynamic diameter whereby 50% of the particles in an aerosol by mass are larger and 50% are smaller [106]. As defined by the WHO, inhalable dust refers to a particle “that can be breathed into the nose or mouth”, which requires a MMAD below 100 µm. They would reach the thoracic region if the MMAD is below 10 µm. The respirable dust is the sub-set that reaches the alveolar region, which requires an MMAD below 4 µm.

A useful concept to simplify the risk assessment of inhalable particles is the concept of poorly soluble low toxicity (PSLT) particles. The PSLT approach addresses inhalable particles, i.e. particles with a MMAD below 10 µm that are not critical fibres (as identified by the WHO criteria explained later on) and moreover have a low solubility in biological fluids and show a low or no inherent toxicity. Such particles are not expected to show a specific toxicity. However, they may show general particle toxicity in particular after chronic exposure to very high doses, which is often explained by "lung overload". The hypothesis of lung overload was proposed about 30 years ago by Paul Morrow [107]. It describes a state where lung clearance by alveolar macrophages is impaired due to continuous high lung doses of PSLT particles (i.e., the macrophages are kind of “saturated” or “full”). When particle exposure continues to be high, lung overload is proposed to lead to chronic inflammation, epithelial hyperplasia and ultimately the development of lung cancer, as observed in rats.

Although the concept is generally recognized by the scientific community, precise definitions of the “characteristics” of PSLT particles are missing. While ‘low solubility’ might be better definable, the demonstration of ‘low toxicity’ remains in particular challenging [108]. The concept has been published with slightly varying characteristics using different names such as “biopersistent granular dusts” [109], “poorly soluble low-toxicity granular particles” [110] or “granular biopersistent particles without known specific toxicity (GBS)” [111]. To reach some consensus an expert workshop involving experts from academia, industry and regulatory authorities was organized in 2020 to discuss the criteria of PSLT particles [112]. The initial step was to identify criteria for particles that have low solubility (poorly soluble particles, PSP), followed by criteria for low toxicity. PSP can be understood as biopersistent particles. Biopersistence needs to consider the persistence of the particle itself in the biological environment (including solubility/dissolution but also considering macrophage-mediated clearance). This includes the release of additives, monomers and oligomers. In terms of retention time, the experts from the workshop proposed a pulmonary retention half-time for PSP of 60–80 days, and suggested TiO_2_ and carbon black as PSP benchmark materials. Furthermore, they also aimed to define low toxicity particles as those that do “not cause more than minimal and transient granulocytic inflammation up to a lung burden causing overload in the rat”. Therefore, low toxicity is suggested to be assessed based on particle reactivity, oxidative stress and (pro-)inflammatory potential.

The EU project GRACIOUS has proposed an IATA to support grouping of nanoparticles following inhalation, which utilizes elements of the PSLT concept [113]. This IATA describes three important decision nodes. Firstly, the dissolution rate of the particles should be assessed in lung lining and in phagolysosomal fluids. Particles can then be categorized as “instantaneously dissolving” (half-time (t_1/2_) < 10 min), “quickly dissolving” (t_1/2_ < 48 h), “partially dissolving” (t_1/2_ > 48 h and < 60 d) or “very slowly dissolving” (t_1/2_ > 60 d) nanoform (NF). These values for the dissolution half-times were suggested by the authors since there are no scientifically recognized cut-off values. For all particles, except those that are instantaneously dissolving, the IATA continues with the assessment of reactivity and inflammatory potential, which for the very slowly dissolving particles is in line with the PSLT concept.

MNPs are very biopersistent and hence can be categorized as “very slowly dissolving” by default. Dissolution data for different MNPs exist for several environmental compartments. For example, PA-6 particles with a diameter of 100 µm have been shown to have a t_1/2_ of 147 years and thermoplastic polyurethane particles of the same size have been shown to have a t_1/2_ of 73 years, both under Central European conditions [93]. Recently, inhaled MPs have been compared to other inhaled microsized particles and knowledge on inhaled MPs has been summarised [114]. Even though MPs might be considered “biopersistent”, care needs to be taken as these particles may release various additive and/or contaminants.

The US EPA is developing a dedicated IATA for PSLT polymer particles, which combines elements of the PLC and PLST concept [70]. This IATA suggests to start with an assessment of whether the substance is a polymer. For this purpose, the OECD test guidelines (TGs) 118 and 119 for the determination of “the Number-Average Molecular Weight and the Molecular Weight Distribution of Polymers” and of “the Low Molecular Weight Content of a Polymer” using gel permeation chromatography (GPC) are recommended [115, 116]. In a second step, it needs to be determined if the polymer particle is respirable. Particles are considered respirable if a minimum 1% of all particles are smaller than 10 µm. Next, one has to assess water extractability according to OECD TG 120 [117] and the dissolution in biological fluids, such as Gambles solution, which is a lung fluid simulant. Furthermore, “reactivity” needs to be considered. The authors understand particles as non-reactive if they are non-cytotoxic and meet the PLC criteria for oligomeric polymer species (oligomer species content should not be more than 5% of < 1000 Da and 2% of < 500 Da). It should be noted, however, that there may be additional test methods to assess “reactivity”, which will be introduced in detail in the POLYRISK risk assessment framework in the last chapter. Finally, if all decision nodes of the US EPA IATA for PSLT polymer particles are answered with “no”, the polymeric particle is considered to be a respirable PSLT polymer.

### The fibre pathogenicity paradigm (FPP)

Many of the MNPs detected in indoor and outdoor air have a fibre morphology [14, 15, 118]. Polymer fibres may originate from textiles, including clothing and upholstery. In a study that investigated three different indoor samples (two from apartments and one from an office), 33% of the fibres in total were identified as polymeric fibres, with PP being the most abundant [13]. The length of the fibres ranged from 50 to 3250 µm. Fibres < 50 µm were excluded from the counting due to the limit of observation but the authors concluded that such fibres may be present in the atmosphere. It is generally assumed that even fibres longer than 100 µm, can be inhaled as long as their diameter is below than 3 µm [119]. This is because their longitudinal axis can be aligned parallel to the flow streamline, minimizing their flow cross-sectional area to that of a particle with a diameter equal to the fibre width. Therefore, the findings of this study indicate that human inhalative exposure to polymer fibres is possible. Evidence for inhalation of polymer fibres was provided already in the late 1990’s when plastic microfibres were found in lung tissue biopsies taken from workers employed in the synthetic textile industry. Fibres were detected under polarized light microscopy in both healthy and neoplastic lung tissue from lung cancer patients [120]. More recent studies with modern analytical techniques, such as µ-FTIR, confirmed the presence of polymer fibres in human lung tissue. The most abundant polymer type detected was PP, which aligns with the predominantly present PP fibres in the atmosphere, as previously described [26, 121]. Overall, there is evidence that humans are exposed to inhalable polymeric fibres that can originate from a variety of textiles.

To assess the human hazard potential of these polymeric fibres, it is reasonable to consider the well-established fibre pathogenicity paradigm (FPP), which was first described for asbestos, leading to severe lung diseases such as chronic inflammation, fibrosis, and lung cancer [122]. The formation of mesothelioma, a specific type of cancer occurring in the pleura, has been associated with the exposure to asbestos [123]. As the relative 5-year survival is about 8 percent in men and 14 percent in women, mesothelioma is among the most fatal cancer types [124].

Meanwhile, the FPP has also been applied to other fibres, which fulfill specific criteria. The first criterion is that the material is respirable with a diameter < 3 µm. The second criterion is bioperistence. Generally, a fibre is considered biopersistent when the mass-based half-life in rats exceeds 40 days [125]. The third criterion refers to the fibre length. Longer fibres (> 5 µm) will not be efficiently cleared by macrophages. They impale macrophages, causing “frustrated phagocytosis” that results in persistent lung inflammation, which over time can lead to fibrosis and lung cancer. Furthermore, the fibres have a high aspect ratio (fibre length in relation to its diameter > 3). Importantly, the FPP is the first morphology-driven toxicity paradigm. This means that the toxicity of so called “critical” fibres is independent of their chemistry and only dependent on their specific fibre morphology. However, the chemical composition has some importance as it influences the biopersistence.

For nanofibres, it was proposed that the FPP needs to be expanded to also consider flexural rigidity. This means that only rigid nanofibres would act as fibres, while fibres below a certain threshold diameter would rather coil up and act as particles. Intensive research has been conducted to investigate the inhalation toxicity of single and multi-walled carbon nanotubes (SWCNT and MWCNT) [122]. Due to numerous available in vivo studies, a diameter threshold (> 30 nm) could be derived for MWCNTs to classify them as rigid [126].

**Fig. 2:**
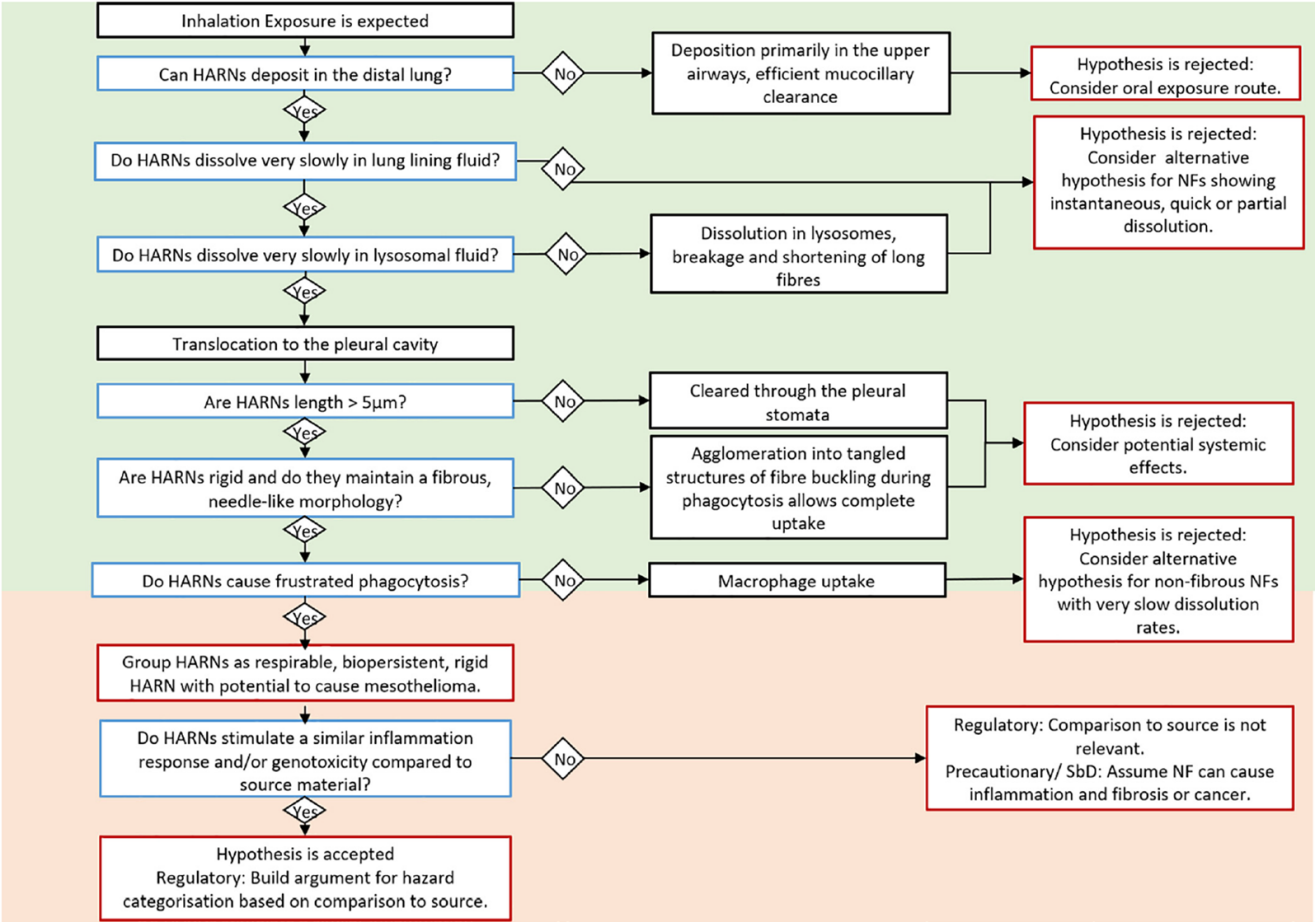
HARN IATA to support the grouping of respirable, biopersistent and rigid HARNs that has been established within GRACIOUS project (taken from Murphy et al. [127])

The EU project GRACIOUS has established an IATA to support the grouping of high aspect ratio nanomaterials (HARN), mainly based on the available knowledge on MWCNTs (Fig. 2) [127]. This IATA is based, in particular, on the AOP 171 (chronic cytotoxicity of the serous membrane leading to pleural/peritoneal mesotheliomas in the rat) and AOP 303 (frustrated phagocytosis-induced lung cancer). It starts with the decision node “Can the HARN deposit in the distal lung?”, which is largely based on size. In the first tier one may use the diameter measured from transmission or scanning electron microscopy (TEM or SEM) measurements as a first proxy while in the second tier MMAD of airborne particles should be assessed. Finally, the third tier suggests the assessment of lung burden in vivo.

Some types of polymer fibres may fulfil the criteria of the FPP. For instance, most of them would be biopersistent. For example, synthetic fibres, including PP, PE and polycarbonate, showed no dissolution, no significant changes to surface area and very slight weight gain following a 180 day in vitro leaching test in physiological fluid (Gamble’s Solution), suggesting that they may persist in vivo [128]. Following the FPP, fibre-shaped polymer particles could have a particular hazard concern. Common polymer fibres have diameters in the range of 0.5–3 µm, which renders them respirable and rigid. However, so far polymer fibres have rarely been tested for effects according to the FPP.

### The POLYRISK risk assessment framework for MNPs: step-by-step

**Fig. 3:**
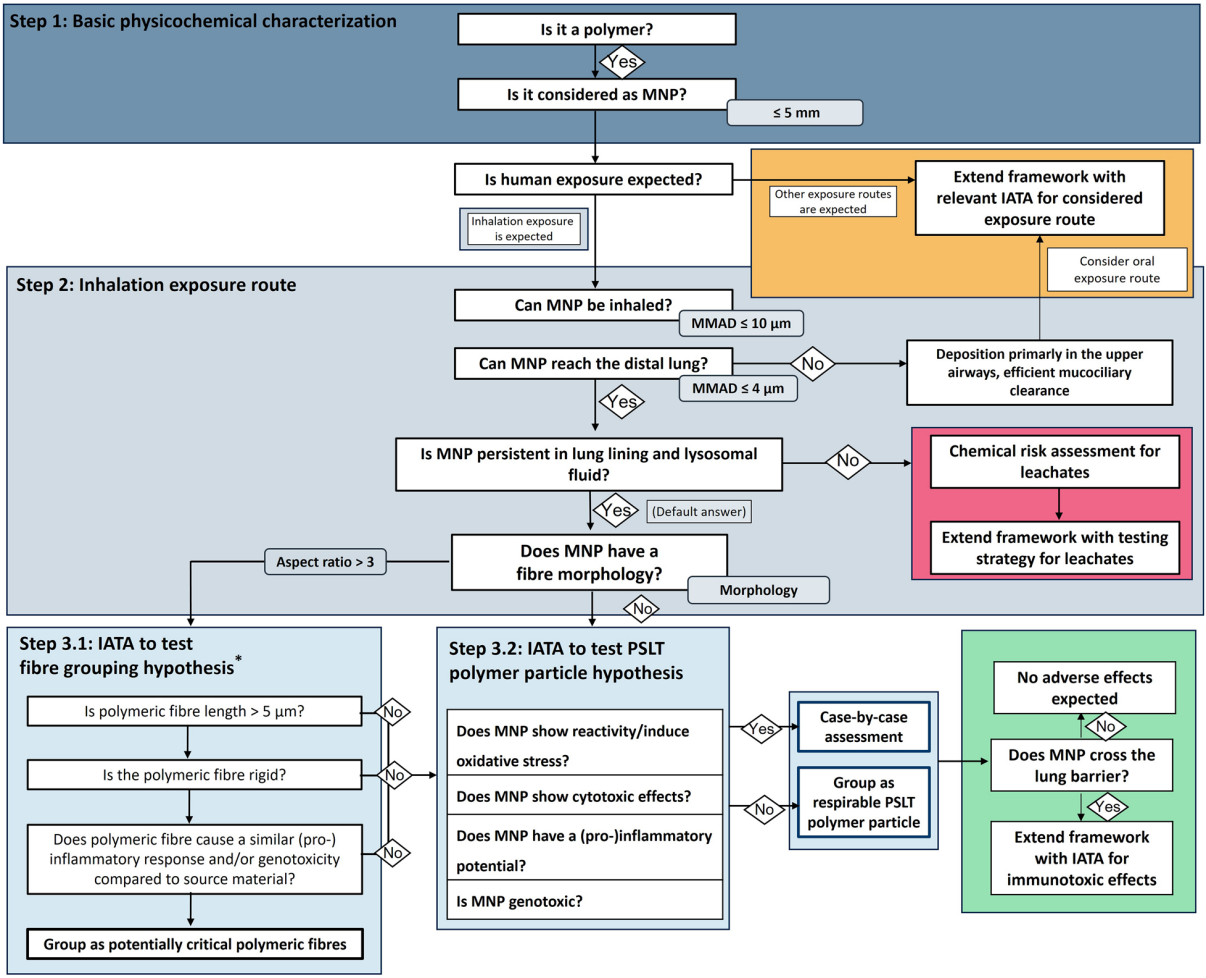
Human health risk assessment framework for MNPs established within the EU POLYRISK project, combining several IATAs. It consists of three main steps that are underpinned by a series of specific questions, which may be answered using the suggested methods. *The IATA for testing the fibre grouping hypothesis (step 3.1) has been slightly adapted from Murphy et al. [127]

The POLYRISK risk assessment framework for MNPs takes into account the suitable elements of the existing state-of-the-art knowledge, approaches and concepts and combines them in a useful manner. It is a modular approach that is based on several IATAs, considering aspects of the PLC concept. As the specific focus is on inhalation, it implements the PSLT concept and the FPP. Due to its modular nature, however, it should be easily amendable. The framework is schematically summarized in Fig. 3.

The framework is modular and shall mainly support classification and prioritization. Importantly, the framework can be entered with a specific polymer particle only or with a mixture/ group of particles- therefore it is applicable for primary and secondary MNPs. Selected methods are included, a comprehensive summary is available in the supplementary information (SI).

### Step 1: Basic physicochemical characterization

In order to determine whether the material of interest falls within the scope of POLYRISK risk assessment framework, an initial basic characterisation of the physicochemical properties should be carried out to answer the following questions:

#### Is the material of interest composed of polymer particles? Can the polymers be classified as low concern?

The initial question is similar to that in the IATA of US EPA [70]. However, it does not only ask whether the particle is made from a polymer. In addition, the *M*_*n*_ should be determined and the presence of low molecular weight species should be assessed, following the PLC concept. *M*_*n*_ can be determined by using GPC as described in OECD TG 118. Low molecular weight species can be determined according to OECD TG 119, which includes monomeric and oligomeric species. As previously explained, according to the PLC concept, the *M*_*n*_ has to be ≥ 1000 Da and oligomers < 1000 Da should not exceed 5% while oligomers < 500 Da should not exceed 2%.

#### Is the material of interest considered as MNPs?

Next, it is important to characterize the size distribution of the material of interest. There are no validated methods specifically for MNPs, however, the OECD TG 125 on "Nanomaterial Particle Size and Size Distribution of Nanomaterials” [129] is considered a very good starting point. This TG gives advice on sample preparation and discusses limitations, including aggregation and agglomeration. It describes eight different methods, including dynamic light scattering (DLS), TEM and SEM. An EM-based approach is regarded the gold standard for quantitative particle size analysis, and in addition it provides information on the shape/ morphology at the same time.

When entering the first step with a complex mixture of MNPs, it might be also helpful to take into account the general recommendations described in the guidance for UVCB substances [68]. This means the aim should be to characterize all components as completely as reasonably possible. For this purpose, it may be helpful to consider the origin as this may provide some initial hints on its likely composition.

#### Is human exposure expected?

Following the basic physicochemical characterization, the framework enquires if human exposure can be expected. As explained in the background, the most relevant exposure routes for humans for MNPs are inhalation and oral uptake. In the following our framework focuses on the inhalation exposure route only. However, the framework is easily amendable with one or more additional IATA(s) specifically for other exposure routes (see yellow box in Fig. 3).

### Step 2: Consideration MNP uptake via inhalation route of exposure

#### Can the MNPs be inhaled and can it reach the distal lung?

Within Step 2, it firstly needs to be assessed whether the particles can be inhaled and reach the distal lung. For this purpose, the MMAD is required. We propose to follow the Guidance Document of the European Commission on the “Determination of Particle Size Distribution, Fibre Length and Diameter Distribution of Chemical Substances” [106]. Suitable methods include cascade impaction, laser scattering/diffraction or the rotating drum method. According to the European Standard EN481, the fraction that can reach the thoracic fraction should have an MMAD of less than 10 µm, while particles that may reach the distal lung should have an MMAD of less than 4 µm [130].

#### Are MNPs persistent in lung lining and/or phagolysosomal fluid?

As a default, MNPs are considered biopersistent. Thus, the default answer would be *yes*. However, as explained before, one also has to consider leaching of chemical additives and/or contaminants. For this purpose, a separate IATA is suggested (see red box in Fig. 3).

#### Proposal for a IATA to test leaching of chemical additives and/or contaminants

Should the investigation of chemical leaching of MNPs be desired, a new IATA can be established (see red box in Fig. 3). We suggest to start with the investigation of the water extractability of the polymer, which is covered by the OECD TG 120 “Solution/ Extraction Behaviour of Polymers in Water” [117]. Furthermore, in order to screen and to quantify further organic components, including the majority of additives, standard analytical methods may be used including sensitive mass spectrometry techniques. For this purpose, the assessment of NIAS in food contact materials may be considered as a suitable starting point, following the comprehensive guidance document published by the International Life Science Institute (ILSI) in 2016 [131]. It provides a detailed overview on the categorisation of NIAS as well as on analytical methods for conducting analytical screening and initial hazard identification. This guidance document also provides helpful advice on how to approach risk assessment of NIAS. The general principles might be equally applicable to assess leaching of chemical additives and/or contaminants from MNPs.

With a focus on the inhalation route of exposure, it should be emphasized that chemical leaching should also be investigated in relevant biological fluids, namely initially in lung lining fluid and then in phago-lysosomal fluid (defined in accordance to ISO 1905/2017 [132]). The analytical screening should be followed by a toxicity screening, which can be conducted using the leachates. For this purpose, the work of Jeong et al. may be useful, which lists relevant KEs that are linked to the 50 most common additives found in MP [84]. The information of possible toxicity mechanisms of theses additives was based on information provided in databases such as US EPA ToxCast™. In general, such databases are very useful for screening possible AOPs (and relevant KEs) associated with additives or other compounds detected in the leachates. Several of these KE can then be assessed using high throughput screening.

It is important to note that there are no specific threshold values for the leaching of chemicals in relevant biological fluids. However, the EU Commission regulation (EU) 10/2011 on plastic materials and articles intended to come into contact with food may be a good starting point for specific threshhold values. The regulation defines specific migration limits (SMLs) for additives and other constituents that should not be transferred to foods. The SMLs can be found in Annex I of this regulation, expressed in mg of substance per kg of food (mg/kg). As there are no threshold values available for the inhalation exposure route, the SMLs provided by the EU regulation (EU) 10/2011 may still serve as a reasonable starting point.

### Step 3-1: IATA to support grouping of polymer fibres according to the FPP

Next, particle morphology, which is available from the physicochemical characterization conducted in step 1, should be assessed to get initial insights whether the FPP might apply. If the particle has an aspect-ratio larger than 3, the fibre IATA suggested by the GRACIOUS project should be followed. This IATA is depicted above (s. Fig. 3) and was published by Murphy and co-authors [127]. There is also a GRACIOUS case study available testing this IATA. In this study, THP-1 macrophage-like cells were chosen as suitable cell culture model [133]. If the MNPs fulfil the criteria for critical fibres according to this IATA, they can be grouped as potentially critical polymer fibres. As the FPP is a morphological paradigm it is then assumed that such a critical polymer fibre will show similar effects as any other critical fibre, and thus, a data rich source material can thus be used for read-across. In terms of exposure, it should be noted that there is an occupational exposure limit to asbestos, as set forth in the Directive 2023/2668/EU, which was recently lowered to 0.01 fibre/cm^3^ [134]. Thus, as a practical outcome it may be concluded that the simple presence of even a low number of potentially critical polymer fibres is sufficient to indicate a severe human health risk. If the MNPs are not fulfilling the criteria of the FPP, they should be tested according to the IATA for PSLT polymer particles.

### Step 3-2: IATA to test PSLT polymer particle hypothesis

The aspect ratio of the polymer particles should be smaller than 3 for a MNP to be considered a particle. As explained above, MNPs are considered poorly soluble by default. Following that step, the MNP should be tested for “low toxicity”. As in our case, the inhalative route is of particular interest, and therefore a suitable lung cell model should be applied. A summary of the most commonly used lung cell models is provided in the SI. When using the POLYRISK risk assessment framework, we recommend the use of human-derived cell culture models, as this framework addresses only human health. For example, there are significant differences between the human and rat respiratory tract.Therefore, using an animal-derived culture model may result in  inappropriate extrapolation of adverse effects for humans [135]. In the SI, we suggest cell culture models which serve as model systems for the lower respiratory tract including alveolar type I and type II cells and are commercially available. Further details are given in the SI.

The screening for “low toxicity” should include cytotoxicity, reactivity/oxidative stress and (pro-)inflammatory responses as those represent most relevant KE for MNPs. In addition, genotoxicity should be investigated as well as this is a very important endpoint on its own, which moreover may be linked to other severe adverse effects. We suggest conducting these screening assays with several types of MNPs so that suitable benchmark materials can be identified. In general, we suggest a tiered in vitro testing strategy beginning with simpler assays and cell models. We also provide some recommendations on suitable assays, however these assays were established for nanoparticles and further adaptations might be necessary. Several of these methods are currently being applied and tested for MNPs within the POLYRISK project. Importantly, for most of the suggested assays standard operation procedures (SOP) for nanomaterials have been published (s. SI). If the MNPs do not show any or only insignificant effects (which might be the case for several MNPs), they can be grouped as PSLT polymer particles. In such a case only very high and prolonged exposure is expected to trigger adverse effects. From known exposure measurements it can be considered unlikely that sufficiently high concentrations of MNPs will be detected over a long time period in indoor and outdoor air. MNPs that can be categorized into this category can therefore be considered of low(er) concern for human health, provided that they would not reach systemic circulation as this would trigger additional testing.

If, however, significant effects in any of the proposed screening assays are observed, a case-by-case assessment for this MNP is required. To date, meaningful data on the toxicity of MNPs are scarce and threshold values cannot yet be derived. Within the POLYRISK project we specifically focus on adverse effects of MNPs on the immune system and a dedicated IATA is currently in progress (see green box in Fig. 3). Suitable in vitro assays for immunotoxicity, as used in the POLYRISK project, are already included in the SI. For all types of MNPs that require a case-by-case assessment, it is also necessary to perform an exposure assessment. A good starting point for the evaluation of the deposition in human airways may be computational models such as the MPPD model or the computational fluid distribution (CFD) model. Both models have been already applied for MNPs to estimate the deposition in the animal lung [136] but also in the human respiratory system [137].

In addition to the toxicological in vitro assays proposed within the framework, in vitro dosimetry models are considered useful. Existing models have already been established for nanomaterials, with the most common examples being the ISD3 model, which is an advanced ISDD (In vitro Sedimentation, Diffusion, Dosimetry) model that additionally considers Dissolution [138], as well as the Distorted Grid (DG) model [139]. There is a publicly available web application based on the DG model provided by the EU project RiskGONE (ID  814425; https://riskgone.wp.nilu.no/) [140]. In addition, the EU project PATROLS (ID 760813) has developed a multi-model graphical user interface, DosiGUI (https://github.com/CentroEPiaggio/DosiGUI). We recommend to apply them for MNPs but care needs to be taken as additional adaptations could be necessary.

In any case, a proper physicochemical characterization of the MNPs is recommended in parallel to the toxicity tests, as often particle properties can change over time or in different media.

## Conclusion

Human exposure to MNPs is inevitable but the assessment of human health risks of MNPs poses several challenges. The lack of reliable methods for both exposure and hazard assessment of MNPs and the lack of meaningful data remain overarching challenges. It is nevertheless obvious that the risk assessment of MNPs cannot be achieved on a case-by-case basis for all the possible particle types in combination with all the possible additives and contaminants. More straightforward approaches are needed that can better cope with this very heterogenous group of particles.

Here we propose a practical, modular risk assessment framework with a particular focus on inhalation as the key exposure route of concern. The framework should be easily amendable, e.g. to also cover other exposure routes. To establish the POLYRISK framework, we implemented existing knowledge and elements of available concepts. It combines different IATAs in a meaningful manner. Further work is currently ongoing underway to specifically address immune-related effects. Moreover, we provide a user-friendly, step-by-step guidance to support the selection of appropriate state-of-the-art methods. They are considered a reasonable starting point for MNPs but may require further adaptations.

Overall, we believe that our risk assessment framework is representing the current state-of-the-art and is an important step forward to support practical risk assessment of MNPs concerning human health. The framework is currently tested in several ongoing case studies within and beyond the POLYRISK project.

## Supplementary Information

Additional file 1.

## Data Availability

Datasets and materials are not applicable to this review.

## Abbreviations

6PPD: *N*-(1,3-Dimethylbutyl)-*N*′-phenyl-p-phenylendiamine
AICIS: Australian Industrial Chemicals Introduction Scheme
AO: Adverse outcome
AOP: Adverse outcome pathway
CEPA: Canadian Environmental Protection Act
DG model: Distorted Grid model
DLS: Dynamic light scattering
EFSA: European Food Safety Authority
FPP: Fibre pathogenicity paradigm
GBS: Granular biopersistent particles without known specific toxicity
GPC: Gel permeation chromatography
HARM: High aspect-ratio materials
HARN: High aspect-ratio nanomaterials
IATA: Integrated approach to testing and assessment
ISD3 model: In vitro Sedimentation, Diffusion, Dosimetry and Dissolution model
KE: Key event
MIE: Molecular initiating event
MMAD: Mass median aerodynamic diameter
M_n_: Number-average molecular weight
MNP: Micro- and nanoplastic particles
MPPD: Multi-path particle dosimetry model
MP: Microplastic particles
MWNT: Multi-walled carbon nanotube
NAM: New approach methodologies
NIAS: Non-intentionally added substances
NP: Nanoplastic particles
OECD: Organisation for Economic Co-operation and Development
PA: Polyamide
PAHs: Polycyclic aromatic hydrocarbons
PCBs: Polychlorinated biphenyls
PDFs: Probability density functions
PE: Polyethylene
PLC: Polymers of low concern
PM: Particulate matter
PM_10_: Particulate matter ≤ 10 µm
PM_2.5_: Particulate matter ≤ 2.5 µm
PP: Polypropylene
PRR: Polymers requiring registration
PS: Polystyrene
PSLT: Poorly soluble low toxicity particle
PSP: Poorly soluble particles
PU: Polyurethane
PVC: Polyvinyl chloride
QA: Quality assurance
QC: Quality control
RFGs: Reactive functional groups
ROS: Reactive oxygen species
SEM: Scanning electron microscopy
SML: Specific migration level
SOP: Standard operation procedure
SWCNT: Single-walled carbon nanotube
t_1/2_: Half-time
TEM: Transmisson electron microscopy
TG: Test guideline
US EPA: United States Environmental Protection Agency
WHO: World Health Organisation

## Glossary

Adverse outcome pathway (AOP): Adverse outcome pathways (AOPs) are conceptual frameworks that outline the sequence of biological events, linking a molecular initiating event (MIE) to an adverse outcome via several key events (KEs). AOPs facilitate the mechanistic understanding of toxicological processes by compiling the existing knowledge in a meaningful manner.
Integrated approach to testing and assessment (IATA): An integrated approach to testing and assessment (IATA) is a flexible approach for chemical safety assessment that “integrates and weights all relevant existing evidence and guides the targeted generation of new data, where required, to inform regulatory decision-making regarding potential hazard and/or risk.
Mass median aerodynamic diameter (MMAD): The mass median aerodynamic diameter (MMAD) is a measure to represent the aerodynamic size distribution of particles. The aerodynamic diameter takes into account particle diameter, particle density, flow velocity and air viscosity. The MMAD represents the value, where half of the mass of the particles in a sample have smaller aerodynamic diameters and half have larger aerodynamic diameters.
Microplastics: There is no harmonized definition. In general, microplastics is used for solid plastic particles being smaller than 5 mm. Microplastics can be further classified into primary microplastics, being intentionally produced in this size range and secondary microplastics, resulting from environmental degradation of larger plastics items (e.g. due to mechanical forces and UV aging).
Nanoplastics: There is no harmonized definition. However, taking into account the definition of nanoparticles, nanoplastics is generally used for plastic particles being smaller than 100 nm. It should be noted that 1000 nm has also been suggested as an upper limit.
Polymer: According to the OECD a ‘polymer’ means a substance consisting of molecules characterized by the sequence of one or more types of monomer units and comprising a simple weight majority of molecules containing at least three monomer units which are covalently bound to at least one other monomer unit or other reactant and consists of less than a simple weight majority of molecules of the same molecular weight [141].
Poor solubility low toxicity particles: The PSLT approach addresses inhalable particles, i.e. particles with a MMAD below 10 µm that are not fibres (meaning they have an aspect ratio of less than 3 µm) that moreover have a low solubility in biological fluids and show a low or no inherent toxicity. Such particles are not expected to show a specific toxicity.
